# Mitral valve replacement with papillary muscle tugging approximation in a patient with severely impaired left ventricular function 14 years after initial mitral valve repair

**DOI:** 10.1093/jscr/rjac339

**Published:** 2022-08-11

**Authors:** Tomoki Nakatsu, Tomonori Shirasaka, Aina Hirofuji, Hiroyuki Kamiya

**Affiliations:** Department of Cardiovascular Surgery, Kushiro Kojinkai Memorial Hospital, Kushiro, Japan; Department of Cardiac Surgery, Asahikawa Medical University, Asahikawa, Japan; Department of Cardiac Surgery, Asahikawa Medical University, Asahikawa, Japan; Department of Cardiac Surgery, Asahikawa Medical University, Asahikawa, Japan

## Abstract

The best treatment for ventricular functional mitral regurgitation still remains unclear. Papillary muscle tugging approximation (PMTA) is a technique known to preserve function of the sub-valvular apparatus, keeping it functionally in synch with the left ventricle systolic and diastolic dynamics. Herein, we present a case of mitral valve replacement with PMTA in a patient with severely impaired left ventricular function 14 years post initial mitral valve repair, which significantly improved during a course of 4 years after the reoperation without any complications.

## INTRODUCTION

In patients with ventricular functional mitral regurgitation (MR), the best treatment option has been unclear. There is less early postoperative mortality post mital valve (MV) repair than MV replacement, but ~30% of patients post repair develop recurrence of MR, and no difference in mid-term survival between repair and replacement [1]. Regarding MV replacement, postoperative impairment of the left ventricular function remains a concern, and mid-term mortality is still high [1, 2]. Therefore, the key to improve survival rate of patients suffering from ventricular functional mitral valve regurgitation lies in finding a better solution for MV replacement while preserving left ventricular function.

Recently, Matsui *et al*. reported a novel MV replacement technique (papillary muscle tugging approximation: PMTA) with good outcome [3]. In this technique, sub-valvular apparatus is completely preserved and approximated papillary muscles are suspended together toward the annulus of the A2 segment. Here, we report a case of MV replacement with PMTA technique in a patient with severely impaired left ventricular function.

## CASE REPORT

**Figure 1:**
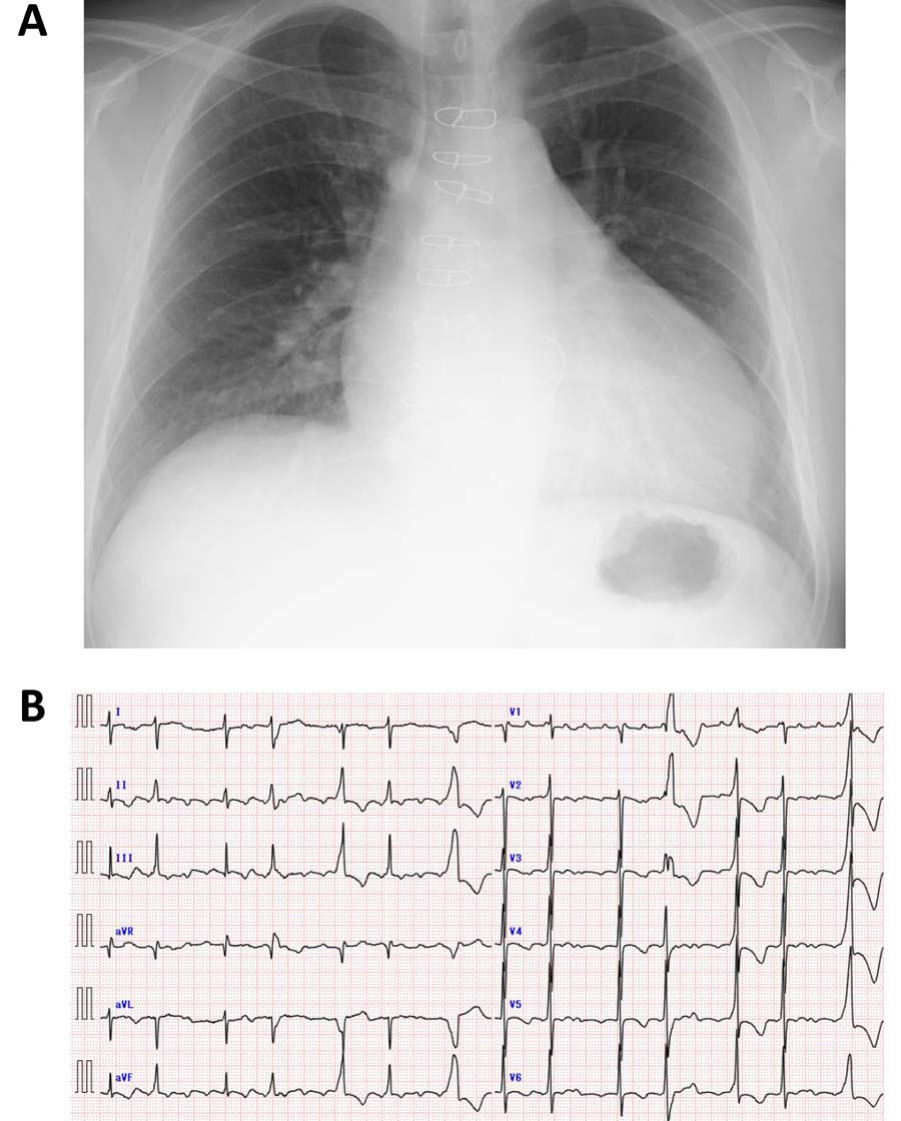
Preoperative chest X-ray (**A**) and electrocardiogram (**B**).

**Figure 2:**
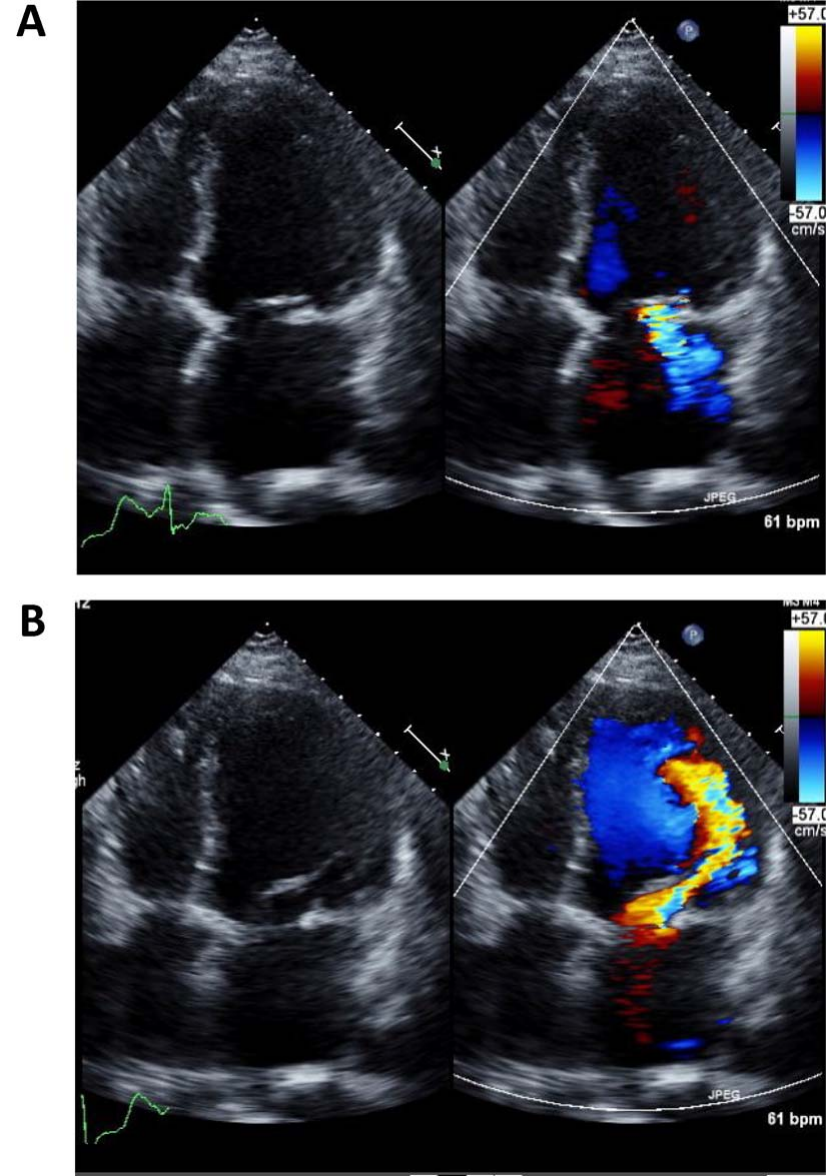
Preoperative echocardiography of left ventricle in systolic (**A**) and diastolic (**B**) phases.

A 50-year-old male who had undergone MV repair 14 years ago due to traumatic mitral valve regurgitation was presented to our department due to heart failure with New York Heart Association (NYHA) III classification. Chest X-ray showed moderate cardiomegaly, and electrocardiogram showed atrial fibrillation with controlled heart rate (Fig. 1A and B). Transthoracic echocardiogram showed moderate MR with severely impaired bi-ventricular function; left ventricular ejection fraction of 20%, (Fig. 2A and B). Coronary angiography showed no significant stenosis. Although indication for mitral valve surgery was marginal [2], surgical intervention was decided to prevent further deterioration of bi-ventricular function.

Operation was performed through re-sternotomy. After cardiac arrest induced by antegrade cardioplegic solution. A right-side left atrial incision was made. Thereafter, the mitral valve was observed: the partial ring-prosthesis did not cover the entire posterior annulus, which had led the whole mitral annuls to significantly dilate. Both mitral leaflets were tethered into the left ventricle.

First, a small incision was created along the anterior leaflet. The anterior chordae were pulled and fixed to each commissure using pledgeted mattress sutures. Then, the papillary muscles were approximated with a 4-0 polypropylene suture. The papillary muscles were suspended toward the annulus of A2 with an expanded polytetrafluoroethylene suture (Gore-Tex CV-3; W. L. Gore & Assoc, Flagstaff, AZ). Mitral valve replacement was then performed at the intra-annular position with a 27-mm mechanical valve (Abbot, St. Paul, MN). Thereafter, tricuspid valve annuloplasty was performed with a 26-mm ring-prosthesis (TriAD, Medtronic, MN), and the aortic clamp was released.

**Figure 3:**
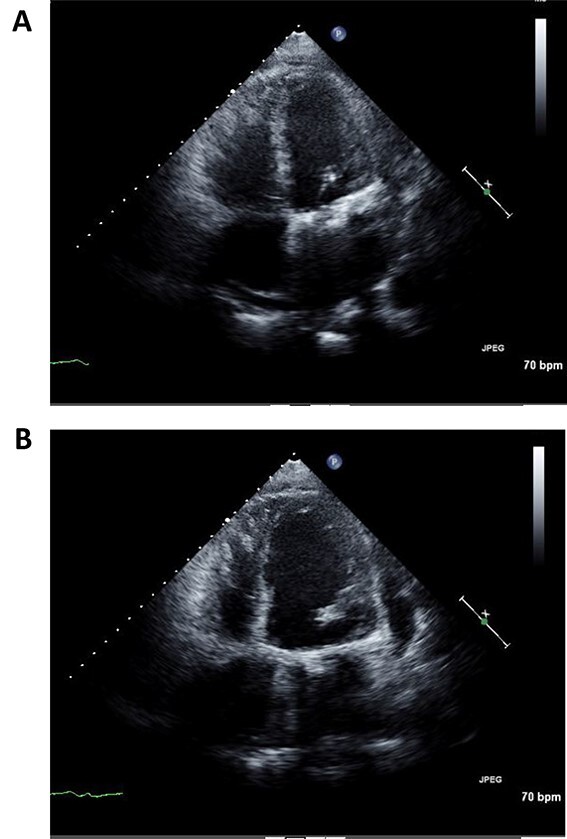
Postoperative echocardiography of left ventricle in systolic (**A**) and diastolic (**B**) phases.

Immediately after the operation, echocardiography revealed improved cardiac function: left ventricular ejection fraction of 37% (Fig. 3A and B). Postoperative course was uneventful and the patient was discharged 2 weeks post operation. Fourth year follow-up revealed further improvement in cardiac function; left ventricular ejection fraction of 42%. The patient continues to do well with NYHA class I status.

## DISCUSSION

Whether MV replacement or repair should be done on patients having ventricular functional MR has been controversial. Recently, Nappi *et al*. reported in their large-scale meta-analysis that MV replacement is associated with increased early mortality but reduced reoperation rate and readmission rate compared with MV repair using annuloplasty in moderate to severe ischemic MR [4]. In general, left ventricular ejection fraction decreases temporary after MV replacement [5], which can be critical in patients with very poor left ventricular function as in the presented case. This may be one of the reasons why MV replacement is associated with increased early mortality [1, 2, 4]. Although preservation of sub-valvular apparatus is a good option, it may not be enough to overcome the initial drop of left ventricular function after MV replacement [1, 4].

As shown in the present case, the PTMA technique may be a reasonable solution to avoid initial drop of LV function after MV replacement. Matsui *et al*. hypothesized the mechanism as follows: Contraction of the papillary muscles causes direct motion of the left ventricular free wall toward the outflow tract owing to transverse connections (tugging effect) in the minor and long-axis directions. Because the position of the papillary muscle heads in an enlarged heart is separated and located backward, the heads should be relocated at the center of the mitral valve ring by approximation and suspension of the heads in the anterior direction, and the turbulent flow that occurs at the ventricular septum after conventional MV replacement can be decreased [3].

The improvement of left ventricular function in our patient may indicate durability of the reverse remodeling effect of the left ventricle provided by the PTMA technique. Matsui’s group reported the results of PTMA technique in 11 patients having non-ischemic functional MR [6]. During their 5-year follow-up, only one patient died, resulting in mid-term survival rate of 90% [6]. Our experience with the present patient supports their findings.

In conclusion, we experienced a case of recurrent MR with very poor left ventricular function in which PTMA technique was highly effective in the course of 4 years. This technique may be effective for such patients with ventricular functional MR. This technique might improve the short-term outcome of MV replacement in patients with ventricular functional MR.

## References

[1] AATS Ischemic Mitral Regurgitation Consensus Guidelines Writing Committee, Kron IL, LaPar DJ, Acker MA, Adams DH, Ailawadi G, et al. 2016 update to the American Association for Thoracic Surgery (AATS) consensus guidelines: ischemic mitral valve regurgitation. J Thorac Cardiovasc Surg 2017;153:e97–e114. 28411753 10.1016/j.jtcvs.2017.01.031

[2] Goldstein D, Moskowitz AJ, Gelijns AC, Ailawadi G, Parides MK, Perrault LP, et al. Two-year outcomes of surgical treatment of severe ischemic mitral regurgitation. N Engl J Med 2016;374:344–53. 26550689 10.1056/NEJMoa1512913 PMC4908819

[3] Matsui Y, Shingu Y, Wakasa S, Ooka T, Kubota S. Papillary muscle tugging approximation for functional mitral regurgitation. Ann Thorac Surg 2019;107:e427–9. 30641062 10.1016/j.athoracsur.2018.11.072

[4] Nappi F, Antoniou GA, Nenna A, Michler R, Benedetto U, Avtaar Singh SS, et al. Treatment options for ischemic mitral regurgitation: a metaanalysis. J Thorac Cardiovasc Surg 2022;163:607–622.e14. 32713629 10.1016/j.jtcvs.2020.05.041

[5] Shafii AE, Gillinov AM, Mihaljevic T, Stewart W, Batizy LH, Blackstone EH. Changes in left ventricular morphology and function after mitral valve surgery. Am J Cardiol 2012;110:403–408.e3. 22534055 10.1016/j.amjcard.2012.03.041 PMC4717321

